# Perioperative Management of Chronic Antithrombotic Agents in Elective Hip and Knee Arthroplasty

**DOI:** 10.3390/medicina57020188

**Published:** 2021-02-23

**Authors:** Daniel C. Santana, Matthew J. Hadad, Ahmed Emara, Alison K. Klika, Wael Barsoum, Robert M. Molloy, Viktor E. Krebs, Michael R. Bloomfield, Nicolas S. Piuzzi

**Affiliations:** 1 Department of Orthopaedic Surgery, Cleveland Clinic, Cleveland, OH 44195, USA; santand2@ccf.org (D.C.S.); hadadm2@ccf.org (M.J.H.); emaraa2@ccf.org (A.E.); klikaa@ccf.org (A.K.K.); molloyr@ccf.org (R.M.M.); krebsv@ccf.org (V.E.K.); bloomfm2@ccf.org (M.R.B.); 2 Healthcare Outcomes Performance Company (HOPCo), Phoenix, AZ 85023, USA; barsouw@ccf.org

**Keywords:** arthroplasty, anticoagulation, venous thromboembolism, major bleeding, surgical complications

## Abstract

Total hip and knee arthroplasty are common major orthopedic operations being performed on an increasing number of patients. Many patients undergoing total joint arthroplasty (TJA) are on chronic antithrombotic agents due to other medical conditions, such as atrial fibrillation or acute coronary syndrome. Given the risk of bleeding associated with TJAs, as well as the risk of thromboembolic events in the post-operative period, the management of chronic antithrombotic agents perioperatively is critical to achieving successful outcomes in arthroplasty. In this review, we provide a concise overview of society guidelines regarding the perioperative management of chronic antithrombotic agents in the setting of elective TJAs and summarize the recent literature that may inform future guidelines. Ultimately, antithrombotic regimen management should be patient-specific, in consultation with cardiology, internal medicine, hematology, and other physicians who play an essential role in perioperative care.

## 1. Introduction

Chronic treatment with antithrombotic agents, including anticoagulants and antiplatelet agents, is a growing component in the management of a variety of medical conditions [1,2,3]. Common indications for the long-term treatment with antithrombotic agents include atrial fibrillation, intrinsic hypercoagulable states, a history of venous thromboembolism (VTE), acute coronary syndrome, and prolonged immobility. Anticoagulants prevent the formation of thrombin in the coagulation cascade, and include vitamin K antagonists (VKAs) (e.g., warfarin), heparin derivatives (unfractionated and low molecular weight heparin (LMWH)), direct thrombin inhibitors (dabigatran), and direct factor Xa inhibitors (rivaroxaban, apixaban, edoxaban). Antiplatelet agents interfere with the adhesion, activation, and aggregation of platelets during thrombus formation; and include cyclooxygenase-1 inhibitors (aspirin) and P2Y12 receptor antagonists (clopidogrel, prasugrel, ticagrelor) [4].

Total hip (THA) and knee (TKA) arthroplasty are common major orthopedic operations. Over 370,000 primary THAs and 680,000 primary TKAs are estimated to have been performed in 2014, with a projected growth of primary THAs by 71% and primary TKAs by 85% by the year 2030 [5]. THAs and TKAs are generally well tolerated procedures but are associated with a higher risk of VTE events [6,7]. Post-operative VTE events are associated with patient morbidity, patient mortality, and increased healthcare costs [8,9,10].

With the growth in the number of patients managed with chronic antithrombotic agents, orthopedic surgeons and their teams will be increasingly tasked to manage perioperative treatment interruption of antithrombotic regimens. Therefore, the purpose of this review is to provide a concise update on the perioperative management of chronic antithrombotic agents during elective THA and TKA based upon published society guidelines and recent trials. This review highlights the need for individualized patient-centered management, and the essential role of working together with internal medicine, vascular medicine, cardiology, pulmonology, and anesthesiology in the multidisciplinary care of increasingly complex surgical patients.

## 2. Overview of Thromboembolism and Bleeding Events after TJA (Total Joint Arthroplasty)

Balancing the risk of adverse thromboembolic events versus major bleeding is the core consideration of perioperative antithrombotic agent management within a TJA. In the pre-operative period, antithrombotic management is primarily targeted to avoid arterial thromboembolism (ATE) predisposed by the patient’s underlying condition(s). In the post-operative period, VTE is of increased concern, which includes deep venous thrombosis (DVT) and pulmonary embolism (PE). Independent risk factors for VTE after a TJA include hypercoagulability, metastatic cancer, stroke, sepsis, obesity, COPD, and HIV infection [11,12,13]. Historically and prior to the routine use of antithrombotic agents, the rate of venography-screened VTE after a TJA had been estimated between 41% and 84% [6,7]. Many of these cases were likely clinically insignificant events. However, with the introduction of antithrombotic agents, the rate of VTE has decreased substantially [14]. Januel et al. report the incidence of symptomatic VTE after THA and TKA with modern antithrombotic use at 0.53% and 1.09%, respectively [15]. While it is clear that the rate of VTE has decreased with routine perioperative antithrombotic agent administration, given the more than 1 million TJAs performed annually, the absolute number of VTE events remains clinically substantial. Notably, even with the increased adoption of perioperative antithrombotic agent use between 1996 and 2010, the rate of pulmonary embolism after TKA had not significantly decreased [16].

On the other hand, major bleeding events after a TJA are also associated with negative patient outcomes and financial ramifications [17,18,19]. While there is no consensus definition for major bleeding after a TJA, it may manifest as hematoma development or external bleeding requiring therapeutic intervention, persistent sanguineous wound drainage, bleeding at a non-surgical site, or need for a post-operative transfusion [20]. Persistent wound drainage or hematoma formation may also increase the risk of surgical site infection [21]. Known patient risk factors for bleeding after a TJA include female sex, pre-operative anemia, BMI < 30, Medicaid insurance, older age, and a higher comorbidity index [22,23,24]. The rate of major bleeding after a TJA with perioperative antithrombic agent administration has been reported between 1.7% and 18%, with variation depending on the definition of bleeding and the potency of antithrombic agents used [20,23,25,26]. Regardless of the agent chosen, pharmacologic VTE prophylaxis increases major bleeding events with only a small or even no effect in reducing mortality from VTE [27]. This brings into question the clinical benefit of reducing rates of VTE with pharmacologic agents [28].

Balancing the risks of thromboembolism versus bleeding becomes more complex still with patients who are prescribed chronic antithrombotic agents, as they typically have comorbidities associated with a higher baseline risk of thromboembolism. The following sections explore evidence-based strategies for the timing of antithrombotic agent interruption and resumption in patients on chronic antithrombotic agents to minimize the risks of thromboembolism and major bleeding during THA and TKA.

## 3. Summary of Guidelines for Perioperative Management of Chronic Antithrombotic Agents

Numerous societies have published guidelines on the management of chronic antithrombotic agents in the perioperative setting, as well as for the purpose of VTE prophylaxis. Common guidelines include those published by the American Academy of Orthopaedic Surgeons (AAOS) in 2010 and 2011 [29,30], the American College of Chest Physicians (ACCP) in 2012 and 2016 [31,32,33], the American College of Cardiology (ACC) in 2017 [34], the American College of Surgeons (ACS) in 2018 [35], the American Society of Regional Anesthesia and Pain Medicine (ASRA) in 2018 [36], and the American Society of Hematology (ASH) in 2019 [37]. Guidelines have also been published outside of the U.S. such as those by organizations in Britain in 2016 [38], Spain in 2018 [39], and France in 2019 [40].

Although the management of chronic antithrombotic agents in the perioperative setting requires separate considerations from the selection of agents for VTE prophylaxis, in our experience, surgeons frequently employ a patient’s previously prescribed chronic antithrombotic agent for VTE prophylaxis post-operatively. There is limited literature to determine whether a management strategy that temporarily employs a different medication than the patient’s chronic agent in the post-operative setting, before switching back to the chronic medication, would enhance post-operative outcomes. Therefore, in this section we summarize and compare the chronic antithrombotic agent management algorithms proposed by existing guidelines, with attention to how to interrupt, bridge, and restart the most commonly encountered medications for the purpose of performing an elective TJA.

### 3.1. Classifying Surgical and Patient Bleeding and Thromboembolism Risk

Most of the guidelines are similar in recommending the classification of surgical timing, surgical bleeding risk, patient bleeding risk, and patient thromboembolism risk before deciding on an antithrombotic agent management plan. The AAOS guidelines classify surgical timing as urgent (surgery necessary within 24 h), semi-urgent (within 24 to 72 h), or elective. We will only be discussing the management of elective TJAs in this review, but in general for urgent and semi-urgent procedures, the reversal of anticoagulation is recommended prior to surgery. We refer the reader to other reviews on the management of antithrombotic agents for hip fracture surgery [41]. For elective TJAs, the AAOS guidelines consider the risk of post-operative hemorrhage for the TJA to be intermediate, which is consistent with other guidelines that suggest it is intermediate or high risk. This classification is largely subjective, but we agree with the AAOS guideline determination. 

After establishing the surgical timing and surgical bleeding risk, the patient can then be stratified based upon their individual thromboembolism and bleeding risk to make specific antithrombotic management decisions. Thromboembolism risk is classified by multiple guidelines as either high, intermediate, or low, typically based upon the patient’s CHADS_2_ [42] or CHA_2_DS_2_-VASc score [43], prior VTE history, the presence of any heart valves or stents, and other medical risk factors (Table 1). The determination of patient bleeding risk post-operatively is more difficult. The ACC recommends considering the patient’s HAS-BLED score [44], which was developed for assessing bleeding risk in the context of atrial fibrillation. The guidelines note that it is predictive of periprocedural bleeding, but not validated for this purpose. Other elements of the patient’s medical history should be considered when assessing the bleeding risk, such as prior bleeding history and laboratory tests (Table 2). Any risk factors should be mitigated to whatever extent possible prior to surgery.

### 3.2. Warfarin Management and Bridging

There is a high degree of consensus among the AAOS, ACCP, ACC, ASRA, and ACS guidelines regarding the perioperative management of warfarin, with minor differences in timing and INR (international normalized ratio) targets. Patients on warfarin undergoing an elective TJA should have warfarin discontinued 5 days before surgery, with a target INR of ≤1.3 according to AAOS, or <1.5 according to ACCP, ASRA, and ACS measured the day before surgery. In refractory cases, vitamin K is recommended as an adjunct to normalize the INR, which typically avoids the need to delay surgery. AAOS recommends oral vitamin K over intravenous (IV) or intramuscular forms; ACCP also recommends oral vitamin K (1 to 2.5 mg), while ACS suggests 1.0 mg IV. The ACC guidelines propose the timing of warfarin interruption based upon INR targets measured 5 to 7 days pre-operatively, interrupting ≥5 days before surgery if the INR is >3.0, 5 days before for INR between 2.0 to 3.0, and 3 to 4 days before for INR 1.5 to 1.9. In any case, individual decisions should be made for patients on higher warfarin maintenance doses or if the INR is known to normalize quickly. According to AAOS, ACCP, ACC, and ACS, warfarin can be resumed at the normal dose the evening of or the morning after surgery if adequate hemostasis has been achieved (12 to 24 h later).

Whether to bridge warfarin with other anticoagulants pre-operatively after its interruption, or post-operatively prior to its re-initiation requires further consideration. The guidelines vary in whether or not they recommend a specific bridging regimen when bridging is indicated. The AAOS guidelines recommend outpatient LMWH for bridging, largely due to its efficacy and cost-effectiveness, unless the patient is pregnant or has renal failure, in which case unfractionated heparin (UFH) is recommended. Other guidelines, such as those by the ACCP, do not recommend specific bridging regimens. According to ACCP, examples of bridging regimens include, for therapeutic dosing: enoxaparin 1 mg/kg BID (twice per day) or 1.5 mg/kg daily; dalteparin 100 IU/kg BID or 200 IU/kg daily; tinzaparin 175 IU/kg daily; IV UFH to achieve an aPTT (activated partial thromboplastin time) target of 1.5 to 2 times control; or SC heparin 250 IU/kg BID without aPTT monitoring; and for prophylactic dosing: enoxaparin 30 mg BID or 40 mg daily; dalteparin 5000 IU daily; or UFH 5000–7500 IU BID.

There is consensus from the AAOS, ACCP, ACC, ASRA, and ACS guidelines that a patient’s thromboembolism risk stratification dictates the need for post-operative bridging for patients on warfarin. For patients with a high risk of thromboembolism (>10% risk per year) and CHA_2_DS_2_-VASc 7–9, bridging, such as with therapeutic LMWH (1 mg/kg BID), is recommended when the INR is subtherapeutic (generally <1.8 according to AAOS; in non-valvular atrial fibrillation, the ACC guidelines recommend bridging when the INR is <2.0). LMWH should be stopped 24 h before surgery, while UFH should be stopped 4 to 6 h pre-operatively. If the patient is also at high bleeding risk, clinical judgment in consultation with vascular medicine, cardiology, and/or anesthesia is recommended regarding the need to bridge, bridging strategy, or if an inferior vena cava (IVC) filter is indicated. Warfarin can be resumed the evening after surgery at the pre-operative maintenance dose; a loading dose is not recommended by the AAOS or ACC guidelines. For intermediate or low bleeding risk, a bridge with therapeutic LMWH can begin when wound hemostasis is achieved (typically within 24 to 48 h of surgery) and stopped when the INR is therapeutic (AAOS recommends INR ≥ 1.8, ACC recommends >2.0 for non-valvular atrial fibrillation). For patients at a high bleeding risk, the guidelines present a few options: a bridge with prophylactic dose LMWH (30–40 mg subcutaneous BID) can be considered according to AAOS, bridging could be delayed to 48 to 72 h post-operatively according to ACS, or in some scenarios it may be better not to bridge according to ACC.

For patients with an intermediate risk of thromboembolism (5% to 10% risk per year) and CHA_2_DS_2_-VASc 5 to 6, the AAOS, ACCP, and ACS guidelines recommend clinical judgement as to whether or not to bridge with therapeutic anticoagulants. The AAOS guidelines suggest that an IVC filter can be considered in this scenario. The ACC guidelines offer more specific guidance, informed in part by the randomized controlled BRIDGE (Bridging Anticoagulation in Patients Who Require Temporary Interruption of VKA Therapy for an Elective Invasive Procedure or Surgery) trial [45]: if the patient is at a high bleeding risk, bridging should typically be avoided, while if they are at a lower bleeding risk but have a history of stroke, transient ischemic attack (TIA), or systemic embolization, bridging is typically indicated. Regardless, in this scenario prophylactic antithrombotic agents are recommended for VTE prophylaxis, such as LMWH, until 12 h prior to surgery and restarted the night of surgery until the INR is therapeutic.

For patients with a low risk of thromboembolism (<5% risk per year) and CHA_2_DS_2_-VASc ≤ 4, bridging is not recommended by the AAOS, ACCP, ACC, or ACS guidelines. Pharmacologic VTE prophylaxis is still indicated as for the intermediate thromboembolism risk group.

The ASRA guidelines apply to surgeries being performed under spinal anesthesia. In some centers, the TJA is performed under spinal anesthesia instead of general anesthesia. These guidelines were first published in 2012 and updated in 2018. Due to the anatomy of the spinal canal, even a small amount of bleeding that results in a spinal hematoma can cause significant morbidity, which makes neuraxial anesthesia a high bleeding risk procedure. For warfarin, the ASRA guidelines recommend an INR target of <1.5 before inserting or removing a spinal catheter. UFH should be discontinued 4 to 6 h prior to a spinal block or prior to catheter removal, with normal coagulation studies verified, similar to TJA. Patients may be re-heparinized one hour after placement or removal. Prophylactic dose LMWH should be discontinued 12 h before and therapeutic dose of LMWH 24 h before catheter insertion or removal, again similar to TJA. The re-initiation of prophylactic LMWH should be delayed at least 12 h after catheter placement and 4 h after removal, while therapeutic LMWH should be delayed 24 to 72 h after catheter placement depending on the bleeding risk.

### 3.3. Direct Oral Anticoagulants

Only the ACC, ACS, and ASRA guidelines discuss the perioperative management of chronic direct oral anticoagulant (DOAC) therapy. DOAC management is typically simpler than the management of warfarin, mainly due to their shorter half-lives, shorter interruption intervals, and because laboratory monitoring is not required. The exact timing for DOAC interruption depends on the agent’s half-life and the patient’s renal function (Table 3). For therapeutic dosing, holding DOACs for a 4 to 5 half-life interval is recommended by the ACC guidelines before beginning an intermediate or high bleeding risk procedure such as a TJA, while a 2 to 3 half-life interval is acceptable for patients on prophylactic dosing. For the re-initiation of DOAC therapy, the ACC recommends waiting 24 to 72 h post-operatively (depending on the patient bleeding risk) and for wound hemostasis to be achieved, at which point therapy can be resumed at the full prior dose, without a loading dose. For low patient bleeding risk where a DOAC is being re-started within 24 h of the procedure, a reduced initial dose can be considered according to the ACC guidelines.

DOAC interruption generally does not require bridging due to the short half-lives of these medications. In the scenario where a patient is at a high post-operative bleeding risk, needs another procedure, or cannot yet tolerate oral medications, the ACC guidelines propose that UFH or LMWH may be temporarily used to bridge to a DOAC, though this is an uncommon scenario.

Most DOACs carry a black box warning for spinal epidural hematoma in the setting of neuraxial anesthesia, lending special consideration for their use in TJAs performed under spinal anesthesia. The ASRA guidelines recommend interruption of Factor Xa inhibitor therapy (apixaban, edoxaban, and rivaroxaban) for 72 h prior to catheter insertion, and that the catheter be removed at least 6 h before the first post-operative dose (Table 3). For dabigatran, a 72-h interruption interval may be used for patients with normal renal function (creatinine clearance (CrCl) ≥ 80 mL/min), but longer intervals are recommended in the setting of reduced renal function (Table 3).

The ASRA guidelines also present interruption intervals for unanticipated procedures, which generally does not apply to elective TJAs. A prior review has suggested that the interruption intervals presented by ASRA for the prophylactic dosing of DOACs in unanticipated neuraxial procedures be used to guide management in TJAs, though we disagree given that an elective TJA is planned and therapeutic DOAC dosing is typically used for chronic anticoagulation [46]. If shorter intervals are being considered for any of the DOACs, a dilute thrombin test (dTT, for dabigatran) or anti-Xa level (for the Factor Xa inhibitors) can be considered.

### 3.4. Anti-Platelet Medications

There is generally consensus among the AAOS, ACCP, ACC, and ACS guidelines on the pre-operative intervals at which to hold aspirin and the antiplatelet agents (clopidogrel, prasugrel, and ticagrelor). For most patients, aspirin should be discontinued 7 to 10 days pre-operatively and the anti-platelet agents 5 to 7 days pre-operatively. The ACCP guidelines recommend that patients at moderate or high cardiovascular risk undergoing surgery continue aspirin perioperatively. Similarly, the ACS guidelines recommend holding aspirin pre-operatively so long as there is no history of percutaneous coronary intervention (PCI).

For patients who have undergone cardiac stenting, surgery should be delayed at least 6 months after a drug-eluting stent and at least 3 months for bare-metal stents, according to the 2010 AAOS guidelines. At this point, aspirin and the antiplatelet agents may be discontinued as above, and restarted when post-operative hemostasis has been achieved.

In the case of procedures performed under spinal anesthesia, the ASRA guidelines recommend a 5 to 7 day pre-operative interruption interval for clopidogrel and ticagrelor, but 7 to 10 days for prasugrel. Antiplatelets can be restarted 24 h post-operatively, or immediately after catheter removal so long as a loading dose is not being used.

## 4. Recent Studies of Perioperative Antithrombotic Agent Management

There have been a number of recent, typically retrospective studies assessing perioperative management protocols for the antithrombotic agents presented previously. Mussa et al. presented a retrospective study of 40 patients on chronic warfarin who underwent THA, half of which continued warfarin perioperatively at a reduced dose and the other half had warfarin held perioperatively [47]. The continuation of perioperative warfarin was associated with a shorter length of stay and a similar rate of post-operative complications and transfusion. Similarly, Phillips et al. published a retrospective study of 61 patients undergoing TKA whose warfarin was continued perioperatively, and found no differences in complication rates or length of stay compared to controls who were not on warfarin [48]. These studies are limited by their small size and retrospective study design; however, these studies suggest that the risk of bleeding associated with continuing warfarin perioperatively may be low. Certainly, further research into the perioperative continuation of warfarin for patients undergoing TJA is necessary.

In evaluation of the ACCP 2012 guidelines, Leijtens et al. presented that 12 out of 13 patients (92%) who received LMWH bridging in their retrospective series of 972 patients undergoing TJA had bleeding complications, with nine (69%) requiring intervention [49]. The ACCP 2012 guidelines, along with guidelines published by AAOS, ACC, and ACS, suggest that bridging should be implemented when the patient’s risk of thromboembolism is high, recognizing that doing so increases the risk of bleeding. This study reminds us that the risks of thromboembolism versus the risks of bleeding have to be carefully considered, ideally in consultation with the multidisciplinary team and the patient. Similar to this study, a Danish cohort study of 649 patients undergoing fast-track TKA or THA on chronic VKA therapy assessed the outcomes of patients who were bridged compared to those who simply had therapy interrupted [50]. This Danish study found no statistically significant difference in VTE or major bleeding events between groups, but there were more bleeding events in the bridged patients (seven events out of 430 patients (1.6%) vs. one event out of 215 patients (0.5%) in the paused group).

There have also been a number of studies assessing perioperative management protocols for the antiplatelet agents, but fewer studies assessing DOACs. A study of 201 patients undergoing TKA found no difference in bleeding complications or perioperative blood loss in 32 patients whose chronic antithrombotic agent was continued perioperatively compared to 169 patients who were not on chronic therapy [51]. Of patients in the continued antithrombotic group, three (9%) were on VKAs, 11 (34%) were on aspirin, 12 (38%) were on antiplatelet agents other than aspirin, six (19%) on DOACs, and one (3%) on both a DOAC and an antiplatelet agent.

Another retrospective study of 757 patients undergoing THA compared outcomes in 205 patients who received aspirin throughout the perioperative period to 552 patients who did not receive any anticoagulants or antiplatelets pre-operatively [52]. This study found no difference in hemoglobin changes, readmission rates, cardiovascular events, or mortality between groups, suggesting that the risks of perioperative aspirin therapy are low. Similarly, Meier et al. presented a study of 175 patients undergoing TJA and found no difference in blood loss or local bleeding complications in the 36 patients who continued aspirin perioperatively compared to 139 who discontinued aspirin [53]. Notably, the group continuing aspirin had higher rates of post-operative knee swelling (81.3% vs. 35.1%).

In addition to studies that have focused on the perioperative continuation of aspirin, the continuation of clopidogrel has been assessed. A study of 142 patients on clopidogrel undergoing TJA found that those who continued clopidogrel perioperatively had higher rates of blood transfusion (31.8% vs. 7.7%, *p* = 0.004) but no difference in cardiac events compared to those whose clopidogrel therapy was interrupted [54]. However, a different study of 67 patients undergoing TKA, 38 of whom continued antiplatelets or vasodilator perioperatively (17 of these patients were on clopidogrel or ticlopidine, and seven on aspirin) found no difference in blood volume loss compared to patients who discontinued these drugs [55]. These studies suggest a higher bleeding risk profile for clopidogrel compared to aspirin, though the bleeding outcome measures used were limited. In our practice, it may be appropriate to continue aspirin therapy perioperatively for select patients, but we typically adhere to society guidelines with the management of other antiplatelet agents. Further research would be necessary to demonstrate that the benefits of continuing antiplatelet agents perioperatively outweigh the risks for certain patients.

## 5. Proposed Algorithm for Perioperative Management of Antithrombotic Agents

**Figure 1:**
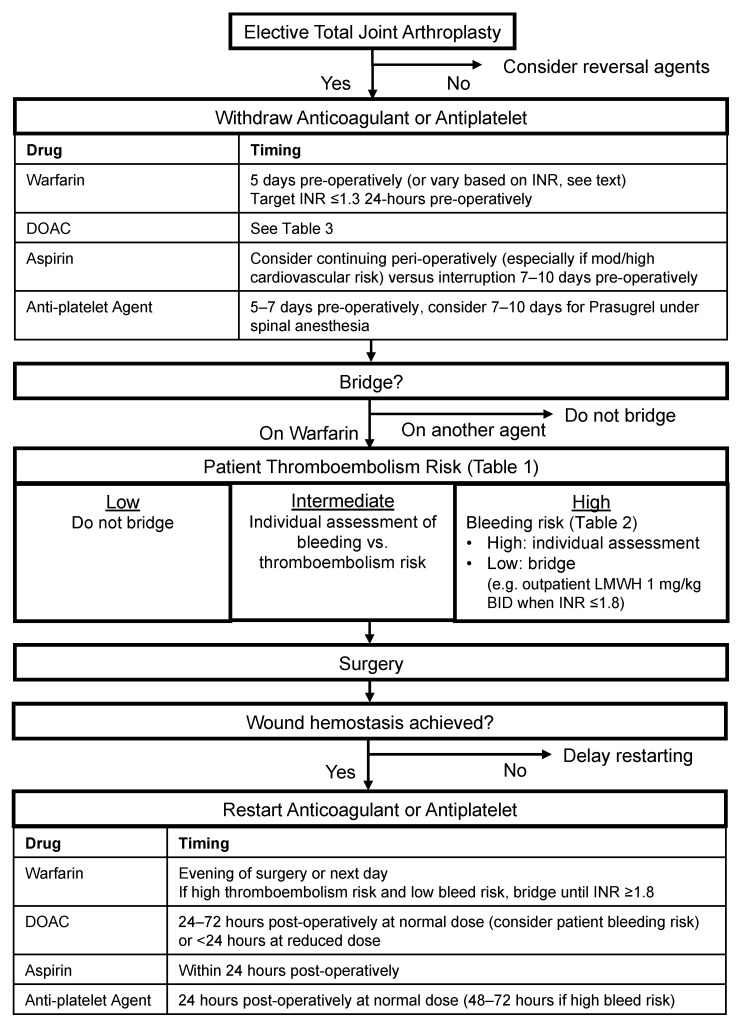
Algorithm for the perioperative interruption of antithrombotic agents. DOAC: direct oral anticoagulant. INR: international normalized ratio. BID: twice per day.

Although much of the recent literature has explored variations in perioperative antithrombotic agent management put forth by society guidelines, with some studies choosing to continue these agents perioperatively, we generally do not feel that there is sufficient evidence to significantly change the management proposed by the guidelines. In exception to this, we do consider continuing aspirin in most patients to be safe. We gather that the recent literature has further highlighted the need to consider individual patient bleeding and thromboembolism risks and make management decisions in concert with other consulting physicians as appropriate. Based upon the guidelines reviewed previously, our perioperative management of antithrombotic agents for TJA is presented in Figure 1.

## 6. Conclusions

The perioperative management of chronic antithrombotic agents in elective TJAs requires careful consideration of patient bleeding and thromboembolism risks. Decision-making as to when to interrupt and restart these agents is based upon the pharmacodynamics of the drugs and the time required for the regeneration of clotting factors. Society guidelines present evidence-based approaches to guide individual patient management decisions, but ultimately management should be a team approach with internal medicine, hematology, cardiology, and other physicians involved in perioperative patient care.

## Figures and Tables

**Table 1:** Patient thromboembolism risk stratification.

Thromboembolism Risk	CHADS_2_ Score *,**	CHA_2_DS_2_-VASc Score ***	Mechanical Heart Valve **,****	VTE History *,**	Medical Risk Factors *,**
High	5 or 6	≥7	Any mitral valve	VTE within 3 months	Stroke or transient ischemic attack within 3 months
			Any caged ball or tilting disc aortic valve	≥2 idiopathic VTE events	Rheumatic valvular heart disease
			Mechanical valve within 3 months		Severe thrombophilia/hypercoagulable state
					Active malignancy
Intermediate	3 or 4	5 to 6	Bileaflet aortic valve with atrial fibrillation or stroke risk factors	VTE within 3–12 months	Non-severe thrombophilia
				Recurrent VTE	
Low	1 or 2	1 to 4	Bileaflet aortic valve without atrial fibrillation and no other stroke risk factors	Previous VTE > 12 months	No history of stroke or TIA

* classified per AAOS (American Academy of Orthopaedic Surgeons) 2010 guidelines [29]; ** classified per ACCP (American College of Chest Physicians) 2012 guidelines [31]; *** classified per ACC (American College of Cardiology) 2017 guidelines [34]; **** classified per ACS (American College of Surgeons) 2018 guidelines [35]. VTE: venous thromboembolism. TIA: transient ischemic attack.

**Table 2:** Considerations for patient bleeding risk.

HAS-BLED Score [44]	Additional Risk Factors *
+1 Hypertension (systolic > 160 mmHg)	Prior bleeding event within 3 months
+1 Abnormal renal function (chronic dialysis, transplant, or SCr ≥ 200 µmol/L)	Platelet abnormality
+1 Abnormal liver function (cirrhosis or significant biochemical derangement)	Supratherapeutic INR
+1 Prior stroke	Prior bleeding from bridge therapy
+1 History of anemia or predisposition to major bleeding	Prior bleeding from similar procedure
+1 Labile INR (<60% of time therapeutic)	
+1 >65 years old	
+1 On antiplatelet agent or NSAID	
+1 Significant alcohol or drug use history (≥8 per week)	
HAS-BLED ≥ 3 is predictive of bleeding events	

* According to ACC 2017 guidelines [34]. INR: international normalized ratio. NSAID: nonsteroidal anti-inflammatory drug.

**Table 3:** Direct oral anticoagulant (DOAC) interruption intervals for TJA (total joint arthroplasty) and spinal anesthesia.

DOAC		Half Life-Normal Renal Function ***	TJA Interruption Interval-Therapeutic Dosing ****		Spinal Anesthesia Interruption Interval-Therapeutic Dosing ‡	
			CrCl (mL/min)	Interval	CrCl (mL/min)	Interval
Direct Thrombin Inhibitor	Dabigatran (Pradaxa)	12–17 h	<15	No data *	<15	Spinal not recommended
			15–29	≥5 days	15–29	
			30–49	≥4 days	30–49	≥5 days
			50–79	≥3 days	50–79	≥4 days
			≥80	≥2 days	≥80	≥3 days
Factor Xa Inhibitor	Apixaban (Eliquis)	12 h	≥30 **	≥2 days	≥30	≥3 days
	Edoxaban (Savaysa)	10–14 h	≥30 **	≥2 days	≥30	≥3 days
	Rivaroxaban (Xarelto)	5–13 h	≥30 **	≥2 days	≥30	≥3 days

* No data, consider dilute thrombin test (dTT); ** no data for creatinine clearance (CrCl) < 30 mL/min, consider anti-Xa level or ≥3 days; *** per individual medication U.S. full prescribing information; **** per ACC 2017 guidelines; ‡ per ASRA 2018 guidelines.

## Data Availability

Data sharing not applicable.
